# Interactions of the eNOS and ACE genes and cigarette smoking in chronic obstructive pulmonary disease

**DOI:** 10.5937/jomb0-34017

**Published:** 2023-01-20

**Authors:** Marija Stanković, Valentina Đorđević, Andrija Tomović, Ljudmila Nagorni-Obradović, Nataša Petrović-Stanojević, Mirjana Kovač, Dragica Radojković

**Affiliations:** 1 University of Belgrade, Institute of Molecular Genetics and Genetic Engineering, Belgrade; 2 Novartis Pharma Services Inc. Representative Office, Sofia, Bulgaria; 3 University of Belgrade, Faculty of Medicine, Belgrade; 4 Clinical Centre of Serbia, Clinic of Pulmonology, Belgrade; 5 Zvezdara University Medical Centre, Department of Pulmonology, Belgrade; 6 University of Belgrade, School of Dentistry, Department of Internal Medicine, Belgrade; 7 Blood Transfusion Institute of Serbia, Belgrade

**Keywords:** COPD, eNOS, ACE, cigarette smoking, genegene interaction, gene-environment interaction, HOBP eNOS, ACE, duvanski dim, GEN-gen interakcija, GEN-sredina interakcija

## Abstract

**Background:**

Chronic obstructive pulmonary disease (COPD) is a complex disorder with unexplained heritability. Interactions of genetic and environmental factors are thought to be crucial in COPD. So, we aim to examine interactions of the endothelial nitric oxide synthase (eNOS) and angiotensin converting enzyme (ACE) genes and cigarette smoking in COPD.

**Methods:**

The eNOS G 894T and ACE ID variants were analyzed in 122 COPD patients and 200 controls from Serbia. The effect of the variants on COPD was assessed by logistic regression. Interactions between eNOS, ACE and cigarette smoking in COPD were evaluated using a case-control model. Interaction between the genes was analyzed in silico.

**Results:**

No effect of the eNOS G 894T and ACE ID variants on COPD was found in our study. Gene-gene interaction between the eN OS T T and A CE D was identified (p=0.033) in COPD. The interaction is realized within the complex network of biochemical pathways. Gene-environment interactions between the eNOS T and cigarette smoking (p=0.013), and the ACE II and cigarette smoking (p=0.009) were detected in COPD in our study.

**Conclusions:**

This is the first research to reveal interactions of the eNOS and ACE genes and cigarette smoking in COPD progressing our understanding of COPD heritability and contributing to the development of appropriate treatments

## Abstract (sr)

**Uvod:**

Hronična opstruktivna bolest pluća (HOBP) je složeno oboljenje sa nerazjašnjenom genetičkom osnovom. Smatra se da su interakcije genetskih i spoljašnjih faktora kljucne u HOBP Stoga je naš cilj bio da ispitamo interakcije gena za endotelijalnu azot-monoksid sintazu (eNOS) i angiotenzin konvertujući enzim (ACE) i duvanskog dima u HOBP

**Metode:**

eNOS G 894T i ACE ID varijante su analizirane kod 122 HOBP pacijenta i 200 kontrola iz Srbije. Uticaj varijanti na HOBP je ispitan logističkom regresijom. Interakcije između eNOS, ACE i duvanskog dima u HOBP su ispitane korišćenjem modela slučaj-kontrola. Interakcija između gena je analizirana in silico.

**Rezultati:**

Prema ovoj studiji eNOS G 894T i ACE ID varijante nemaju uticaj na HOBP GEN-gen interakcija između eNOS T T i ACE D je identifikovana (p=0,033) u HOBP Ova interakcija se ostvaruje u okviru složene mreže biohemijskih puteva. GEN-sredina interakcije između eNOS T i duvanskog dima (p=0,013), i ACE II i duvanskog dima (p=0,009) su detektovane u HOBP u ovoj studiji.

**Zaključak:**

Ovo je prvo istraživanje u kome su otkrivene interakcije između eNOS i ACE gena i duvanskog dima u HOBP što doprinosi našem razumevanju genetičke osnove HOBP i razvoju adekvatnog tretmana.

## Introduction

COPD is already the third leading cause of death worldwide, though it was not predicted to occur until 2030 by the World Health Organization [1]. Despite growing recognition, as an important international health problem, COPD is still underrecognized, underdiagnosed and undertreated although defined as a preventable and treatable disorder by the GOLD standard [1] [2].

COPD is featured by abnormal inflammation of the lungs in response to inhalation of noxious particles or toxic gases, such as cigarette smoke [2]. The disease is characterized by four structural lesions including emphysema, small airway remodeling, vascular remodeling with pulmonary hypertension and mucus overproduction and chronic bronchitis and one functional lesion - acute exacerbation [3]. Complex etiology and high heterogeneity of COPD, have made the mechanisms of pathogenesis difficult to unravel precluding the progress in the development of safe and effective treatments [4].

After a long break, a part of the genetic component in the occurrence of COPD has been upgraded beyond 1-antitrypsin, with the genes for 1 nicotinic acid receptor, hedgehog interacting protein, Rho GTPase activating protein, etc. Nonetheless, so far identified genetic variants can explain only a small part of estimated COPD heritability [5]. Actually, socalled »missing heritability« of COPD has been suggested to be hidden in unidentified interactions of genetic and environmental component. Moreover, cigarette smoking, as an essential risk for COPD, can be particularly detrimental in interaction with genetic component [6].

Cigarette smoking can also cause vascular endothelial dysfunction that can occur even before COPD [7]. Recent studies have pointed out that endothelial dysfunction underlies pulmonary hypertension and emphysema caused by pulmonary vascular remodeling and loss of lung tissue, respectively [7]. Two genes, eNOS and ACE have a crucial effect on endothelial function but also play a role in inflammation, fibrosis, oxidative stress and repair in the lungs [8] [9].

The eNOS G894T variant (rs1799983), located in exon 7 resulting in Glu298Asp change, was reported to reduce eNOS transcriptional activity and NO production in endothelial cells showing 63% and 20% activity in GT and TT carriers, respectively [10] [11]. Produced mainly by endothelium, NO has protective effects on vascular system by promoting endothelial homeostasis, inhibiting platelet aggregation and suppressing the release of inflammatory mediators [7]. To date, a link between the G894T variant and COPD or pulmonary hypertension in COPD was discovered in Tunisians and Chinese, but not in Indian population and Australians [8] [10] [12] [13] [14]. Also, available meta-analyses of essential hypertension found no connection of the G894T variant in Caucasians, in contrast to Asians, where more precisely the connection was observed in southern but not in northern Han Chinese [15] [16].

An insertion (I) or deletion (D) of Alu repeat sequence within the intron 16 of the ACE gene, denoted as ID variant (rs1799752), is accounted for 47% of the total variance of serum ACE, and its relation to hypertension has been intensively studied. The II, ID and DD genotypes are associated with the lowest, intermediate and highest ACE levels, respectively [9]. ACE plays a central role in rennin-angiotensin system through generation of a powerful vasoconstrictor angiotensin II and kallikrein-kinin system by degrading a vasodilator bradykinin [9] [17]. However, the effect of the ID variant on essential or pulmonary hypertension is very small. A recent, comprehensive meta-analysis found no connection of the ID variant with COPD and pulmonary hypertension in COPD overall, but subgroup analysis did reveal the link of the D variant and COPD and associated pulmonary hypertension in Asians [18].

Interactions of genetic and environmental factors have been insufficiently investigated in COPD, though they are expected to have crucial effect in complex diseases. On the other side, exact contribution of endothelial dysfunction to the pathogenesis of COPD, is incompletely understood. That was an impetus for us to comprehensively examine interaction between multifunctional genes eNOS and ACE and cigarette smoking, which has not been addressed in COPD, so far. The aim of our research was to examine the effects of eNOS G894T and ACE ID variants on the pathogenesis of COPD in Serbian population. Gene-gene and gene-environment interactions between eNOS, ACE and cigarette smoking were assessed in COPD using a case-control model and *in silico *analysis.

## Materials and methods

### Case-control design

The study is conducted on patients with COPD and control subjects with normal lung function (NLF controls) recruited from 2002 to 2010, from University Clinical Center of Serbia and Zvezdara University Medical Center, and voluntary blood donors (VBD controls) recruited from 2000 to 2002, from Blood Transfusion Institute of Serbia. Patient group consisted of 122 subjects with COPD diagnosis established according to GOLD standard [2]. NLF controls encompassed 100 subjects without clinical evidence of COPD and with normal pulmonary function measurements. Medical tests performed and inclusion criteria for COPD patients and NLF controls are given elsewhere [6]. VBD controls included 100 subjects without evidence of any disease and with hematological parameters within the reference range. Subjects originate from the same geographic area and are unrelated. The study was approved by the local Ethics Committees including: Ethical Committee of Zvezdara University Medical Center (from 25.2.2009) and the IMGGE (Institute of Molecular Genetics and Genetic Engineering) Ethical Committee (O-EO-004/2015/2 from 31.3.2016 and OEO-022/2020 from 16.9.2020). Informed consent was obtained from each participant. The study was in accordance with the Helsinki Declaration.

### Determination of genotypes

Genomic DNA was extracted from whole blood using a GFX Genomic Blood DNA Purification Kit (Amersham Biosciences).

The eNOS G894T variant was distinguished using the PCR-RFLP method as previously published [19].

Detection of the ACE ID variant was enabled using the PCR method as previously described [20]. Genotypes were scored without knowledge of the sample phenotypes by two independent observers.

### Statistical analysis

Data are expressed as a percentage or mean ± standard error of mean (SEM). Deviation of genotype distribution from the Hardy–Weinberg equilibrium was assessed by X^2^-test. A comparison of variables was performed using the X^2^-test, Fisher’s exact test or the Mann–Whitney U-test, as appropriate.

The effect of genotypes and alleles on the pathogenesis of COPD was assessed by the odds ratio (OR) with the 95% confidence interval (CI) calculated by binary logistic regression. The outcome variable was adjusted for potential confounding factors such as age, gender and smoking status.

Interaction of genetic and environmental factors was estimated by calculating the effect of each risk factor and their joint effect, relative to reference category, in a case-control model as proposed by Ottman [21]. Interaction is estimated on a multiplicative scale as the departure of joint effects from multiplicative ORs [22] [23]. The analysis was performed under dominant (AA *versus* Aa and aa genotype) and recessive (AA and Aa *versus *aa genotype) models.

A p-value of less than 0.05 was considered significant. Adjustment for multiple testing was notapplied, as analyzed variants are a part of pre-established hypothesis [24]. Statistical analysis was performed using the Statistical Package for the Social Sciences (SPSS, Version 20).

### Analysis of gene-gene interaction within the network

Interaction between the eNOS and ACE genes with other genes in the network was explored using a web-based tool GeneMANIA [25]. The network was created based on physical, genetic and predicted interactions, pathways and co-localization using automatic weighting option. GeneMANIA was accessed on 23 August 2021.

## Results

The group of COPD patients and NLF controls were primarily compared in order to identify geneticvariants and interactions of genetic and environmental factors implicated in the pathogenesis of COPD, while VBD controls were introduced for additional testing of gene-gene interaction since no carrier of the double risk genotype, eNOS TT and ACE DD, was detected by using NLF controls. Basic characteristics and clinical parameters of COPD group and control subjects are showed in Table 1. The differences in age, gender, and smoking status among groups were used for adjustment of the outcomes.

**Table 1:** Characteristics of COPD and control groups. COPD-chronic obstructive pulmonary disease, NLF-normal lung function controls, VBD-voluntary blood donor controls, N-number of subjects, FEV1-forced expiratory volume in 1 second, FVC-forced vital capacity.<br>Comparison between patients and control groups ap<0.05, bp<0.001.

	COPD	NLF	VBD
Subjects (N)	122	100	100
Age of disease onset/age (years)	45.0±1.6	50.9±1.4^a^	39.7±1.0^a^
Gender (male, %)	70.2	35.0^b^	84.8^a^
Smokers (%)	69.7	50.0^a^	–
Smoking history (pack-years)	27.4±1.7	26.3±2.3	–
FEV_1_ (% of predicted)	45.3±2.3	110.1±1.7^b^	–
FEV_1_/FVC	62.4±2.0	98.3±0.9^b^	–

The genotype distributions were not in the Hardy–Weinberg equilibrium for the eNOS G894T variant in VBD group encompassing healthy subjects. The observed distribution of G894T genotypes was different than expected (X^2^=4.04, df=1, p<0.05) with a slight increase of GT genotype and a decrease of TT genotype in VBD controls. The disequilibrium could be explained by unfavorable effects of the T allele on health status [26].

There was no difference in the distribution of eNOS G894T and ACE ID alleles and genotypes among COPD group and NLF controls nor among COPD group and VBD controls, Table 2. Also, theeNOS and ACE variants showed no influence on severity and early onset of COPD (Table 3).

**Table 2:** Distribution of eNOS and ACE gene variants in COPD patients and controls. COPD-chronic obstructive pulmonary disease, NLF-normal lung function controls, VBD-voluntary blood donor controls, *p*-p value, OR-odds ratio, CI-confidence interval, N-number of subjects, eNOS-endothelial nitric oxide synthase, ACE-angiotensine converting enzyme.<br>^a^adjusted for age, sex and smoking status, ^b^adjusted for age and sex.

Gene, model	COPD, %	NLF, %	VBD, %	OR (95% CI), p	
Genotype/allele	N=122	N=100	N=100	COPD *versus *NLF^a^	COPD versus VBD^b^
eNOS G894T genotypes and allele					
GG	43.4	49.0	37.0	1	1
GT	41.8	38.0	55.0	1.39 (0.73–2.68), 0.316	0.68 (0.38–1.25), 0.216
TT	14.8	13.0	8.0	1.30 (0.52–3.29), 0.575	1.53 (0.58–4.05), 0.394
T	35.7	32.0	35.5	1.23 (0.78–1.92), 0.374	1.02 (0.67–1.53), 0.936
eNOS G894T dominant model					
GG	43.4	49.0	37.0	1	1
T	56.6	51.0	63.0	1.37 (0.75–2.12), 0.306	0.79 (0.45–1.41), 0.432
eNOS G894T recessive model					
G	85.2	87.0	92.0	1	1
TT	14.8	13.0	8.0	1.12 (0.47–2.69), 0.801	1.89 (0.75–4.72), 0.175
ACE ID genotypes and allele					
II	24.6	23.0	26.0	1	1
ID	47.5	48.0	55.0	0.63 (0.29–1.37), 0.241	1.36 (0.67–2.77), 0.400
DD	27.9	29.0	19.0	0.62 (0.26–1.46), 0.276	1.95 (0.84–4.54), 0.121
II	51.6	53.0	46.5	0.79 (0.52–1.22), 0.291	1.35 (0.91–2.02), 0.136
ACE ID dominant model					
II	24.6	23.0	26.0	1	1
D	75.4	77.0	74.0	0.63 (0.30–1.30), 0.207	1.52 (0.77–2.99), 0.226
ACE ID recessive model					
I	72.1	71.0	81.0	1	1
DD	27.9	29.0	19.0	0.83 (0.42–1.66), 0.606	1.58 (0.80–3.14), 0.190

**Table 3:** eNOS and ACE variants in severity and early onset of COPD. COPD-chronic obstructive pulmonary disease, FEV_1_-forced expiratory volume in 1 second, *p*-p value, OR-odds ratio, CI-confidence interval, N-number of subjects, eNOS-endothelial nitric oxide synthase, ACE-angiotensine converting enzyme.<br>^a^crude, ^b^adjusted for smoking status and sex.

Gene, model	FEV1, % pred		OR (95% CI), *p* ^a^	COPD onset, years		OR (95% CI), *p* ^b^
Genotype/allele	<50, %<br>N=82	50, %<br>N=40		<40, %<br>N=34	40, %<br>N=88	
eNOS dominant model						
GG<br>T	49.4	33.3	1	31.2	47.0	1
	50.6	66.7	0.51 (0.23–1.14), 0.101	68.8	53.0	2.36 (0.88–6.33), 0.087
eNOS recessive model						
G<br>TT	86.1	82.1	1	81.2	86.7	1
	13.9	17.9	0.74 (0.26–2.08), 0.568	18.8	13.3	2.49 (0.74–8.43), 0.141
ACE dominant model						
II<br>D	25.3	20.5	1	25.0	24.1	1
	74.7	79.5	0.76 (0.30–1.93), 0.565	75.0	75.9	0.94 (0.32–2.81), 0.919
ACE recessive model						
I<br>DD	74.7	69.2	1	75.0	73.5	1
	25.3	30.8	0.76 (0.33–1.78), 0.531	25.0	26.5	0.56 (0.18–1.69), 0.301

The frequency of the double risk genotype, eNOS TT and ACE DD, was found to be 4.1% in COPD group, while there was no carrier (0.0%) of such a genotype in NLF controls and VBD group, Table 4. The eNOS TT and ACE DD genotype was significantly higher when COPD patients were compared with both control groups (4.1% *versus *0.0%, p=0.0074). Moreover, gene-gene interaction among eNOS and ACE was identified under the dominantrecessive model, between COPD and VBD group as showed in Table 4. Significantly higher frequency of the double risk genotype, eNOS TT and ACE D, was detected in COPD patients than in VBD controls (11.5% *versus *4.0%, p = 0.033). The double risk genotype of eNOS and ACE increased the risk factor 4.21-fold for COPD, as compared to the reference genotype, eNOS G and ACE II. Interaction between the eNOS TT and ACE D variants was more than multiplicative (0.69×1.29<4.21), showing the genes acting within the same pathway [23].

**Table 4:** Gene-gene interactions between eNOS and ACE in COPD. COPD-chronic obstructive pulmonary disease, NLF-normal lung function controls, VBD-voluntary blood donor controls, *p*-p value, OR-odds ratio, CI-confidence interval, N-number of subjects, eNOS-endothelial nitric oxide synthase, ACE-angiotensine converting enzyme.<br>^a^adjusted for age, sex and smoking status, ^b^adjusted for age and sex.

Gene-gene interaction	COPD, %	NLF, %	VBD, %	OR (95% CI), *p*	
Genotype/allele	N=122	N=100	N=100	COPD versus NLF^a^	COPD versus VBD^b^
eNOS - ACE, dominant model					
GG-II	10.7	12.0	13.0	1	1
T-II	13.9	11.0	13.0	2.09 (0.59–7.36), 0.250	1.52 (0.49–4.76), 0.469
GG-D	32.8	37.0	24.0	0.83 (0.29–2.36), 0.727	2.61 (0.93–7.29), 0.068
T-D	42.6	40.0	50.0	1.01 (0.36–2.81), 0.990	1.60 (0.61–4.21), 0.337
eNOS - ACE, recessive model					
G-I	61.5	58.0	73.0	1	1
TT-I	10.6	13.0	8.0	0.77 (0.28–1.93), 0.538	1.59 (0.60–4.19), 0.349
G-DD	23.8	29.0	19.0	0.72 (0.34–1.40), 0.300	1.46 (0.72–2.98), 0.296
TT-DD	4.1	0.0	0.0	0.999	0.999
eNOS - ACE, dominant - recessive model					
GG-I	32.8	32.0	32.0	1	1
T-I	39.3	39.0	49.0	1.07 (0.53–2.18), 0.846	0.87 (0.46–1.65), 0.665
GG-DD	10.7	17.0	5.0	0.55 (0.21–1.46), 0.231	2.54 (0.71–9.14), 0.152
T-DD	17.2	12.0	14.0	1.42 (0.53–3.79), 0.486	1.15 (0.48–2.76), 0.762
eNOS - ACE, recessive - dominant model					
G-II	21.3	19.0	22.0	1	1
TT-II	3.3	4.0	4.0	0.59 (0.09–3.86), 0.583	0.69 (0.14–3.34), 0.641
G-D	63.9	68.0	70.0	0.56 (0.26–1.22), 0.146	1.29 (0.63–2.65), 0.488
TT-D	11.5	9.0	4.0	0.76 (0.24–2.42), 0.644	4.21 (1.12–15.73), 0.033

**Figure 1:**
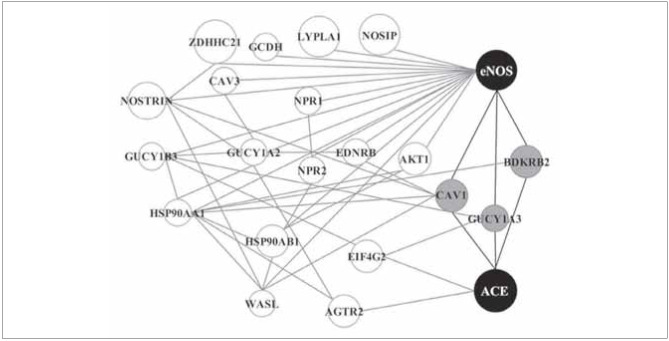
The biochemical pathways of eNOS and ACE interaction. Figure 1 eNOS and ACE (black circles) interact within the network of biochemical pathways (white circles). The network is based on physical, genetic and predicted interactions, pathways and co-localisation using a web-based tool GeneMANIA. The simplest interaction is realised by caveolin-1, GUCY1A1 and BDKRB2 (grey circles). The larger size circles indicate the genes with the greater degree in the network.<br>ACE-angiotensin converting enzyme, ACE2-angiotensin converting enzyme 2, AGTR2-angiotensin II receptor type 2, AKT1-AKT serine/threonine kinase 1, ATP6AP2-ATPase H+ transporting accessory protein 2, BDKRB2-bradykinin receptor B2, CAV1-caveolin-1, CAV3-caveolin-3, EDNRB-endothelin receptor type B, EIF4G2-eukaryotic translation initiation factor 4 gamma 2, eNOS-endothelial nitric oxide synthase, GUCY1A2-guanylate cyclase 1 soluble subunit alpha 2, GUCY1A1-guanylate cyclase 1 soluble subunit alpha 1, GUCY1B1-guanylate cyclase 1 soluble subunit beta 1, HSP90AB1-heat shock protein 90 alpha family class B member 1, LYPLA1-lysophospholipase 1, MYH9-myosin heavy chain 9, NOSIP-nitric oxide synthase interacting protein, NOSTRIN-nitric oxide synthase trafficking, NSMAF-neutral sphingomyelinase activation associated factor, REN-renin, ZDHHC21-zinc finger DHHC-type containing 21.

Exploring gene-gene interaction of the eNOS and ACE genes using a web-based tool GeneMANIA revealed their connection within a complex network of biochemical pathways showed in Figure 1. However, the closest link between eNOS and ACE is realized by bradykinin receptor B2 (BDKRB2), guanylate cyclase 1, soluble, alpha 1 (GUCY1A1) and caveolin-1 (Figure 1).

Gene-environment interactions of eNOS and ACE variants indicated no impact of the risk alleles, asa single risk factors, while the effect of cigarette smoking, as a single risk factor, showed significant effect on COPD by all tested models, Table 5. Gene-environment interaction between eNOS and cigarette smoking was identified under the dominant model. Significantly higher frequency of carriers of risk allele the eNOS T, smokers, was detected in COPD group than in NLF controls (40.9% *versus *25.0%, p = 0.013). The T variant and cigarette smoking interaction increased the risk 3.37-fold for COPD, as compared to the reference category, GG and non-smokers. Interaction between the T variant and smoking was less than multiplicative (2.16x3.11>3.37) [22] [23].

**Table 5:** Gene-environment interactions between eNOS, ACE and cigarette smoking in COPD. COPD – chronic obstructive pulmonary disease, NLF – normal lung function controls, p-p value, OR – odds ratio, CI – confidence interval, N – number of subjects, eNOS – endothelial nitric oxide synthase, ACE – angiotensine converting enzyme.<br>^a^adjusted for age and sex.

Gene-environment interaction, model		COPD, %<br>N=122	NLF, %<br>N=100	OR (95% CI), *p* ^a^
Genotype/allele	cigarette smoking			
eNOS – smoking, dominant model				
GG	absent	9.6	24.0	1
T	absent	16.5	26.0	2.16 (0.75–6.20), 0.152
GG	present	33.0	25.0	3.11 (1.17–8.23), 0.022
T	present	40.9	25.0	3.37 (1.29–8.80), 0.013
eNOS – smoking, recessive model				
G	absent	23.5	44.0	1
TT	absent	2.6	6.0	0.94 (0.19–4.78), 0.944
G	present	61.7	43.0	2.03 (1.02–4.05), 0.043
TT	present	12.2	7.0	2.45 (0.80–7.51), 0.117
ACE (D) – smoking, dominant model				
II	absent	6.0	16.0	1
D	absent	20.0	34.0	1.71 (0.37–3.68), 0.787
II	present	18.3	7.0	4.93 (1.31–18.57), 0.018
D	present	55.7	43.0	1.93 (0.65–5.72), 0.233
ACE (D) – smoking, recessive model				
I	absent	14.7	37.0	1
DD	absent	11.5	13.0	1.85 (0.64–5.38), 0.259
I	present	58.2	34.0	3.06 (1.40–6.70), 0.005
DD	present	15.6	16.0	1.58 (0.58–4.27), 0.367
ACE (I) – smoking, dominant model				
DD	absent	11.5	13.0	1
I	absent	14.7	37.0	0.54 (0.19–1.57), 0.259
DD	present	15.6	16.0	0.85 (0.27–2.68), 0.788
I	present	58.2	34.0	1.65 (0.63–4.35), 0.308
ACE (I) – smoking, recessive model				
D	absent	20.0	34.0	1
II	absent	6.0	16.0	0.85 (0.27–2.68), 0.787
D	present	55.7	43.0	1.65 (0.78–3.47), 0.186
II	present	18.3	7.0	4.21 (1.43–12.40), 0.009

Given that the frequency of ACE I and D alleles vary from 0.4–0.6 between races, interactions ofboth, the I and D, variant and cigarette smoking were tested, Table 5 [27]. Only the I allele showed interaction with cigarette smoking under the recessive model. Significantly more carriers of risk genotype the ACE II, smokers, was found in COPD than in NLF controls (18.3% *versus *7.0%, p = 0.009). Gene-environment interaction between the II genotype and cigarette smoking increased the risk 4.21-fold for COPD, as compared to the reference, ACE D and non-smokers. Interaction between the I variant and smoking was more than multiplicative (0.85x1.65 <4.21).

## Discussion

Our results (Table 2 and Table 3) showed no association of the eNOS G894T and ACE ID variants with the pathogenesis of COPD in Serbian population. Our results also bring the first data for the G894T variant in COPD for European Caucasians, which are in addition, scarcely available and controversial [8] [10] [11] [12] [13].

eNOS produces NO which influences the reduction of smooth muscle tone, inhibition of cell proliferation and migration and suppression of the release of inflammatory mediators [7]. Expression of eNOS is found to be reduced in lungs of patients with severe COPD and the 894T variant is linked with reduced levels of NO in plasma [10] [11] [12] [13] [28]. Also, the expression levels of eNOS are correlated with alveolar repair and vascular regeneration [29]. Thus, lower activity of eNOS, linked with the T variant, could negatively affect these processes causing endothelial dysfunction.

ACE is highly expressed in the lungs and primarily operates as a powerful vasoconstrictor as it generates angiotensin II and also disposes vasodilator bradykinin [9] [17]. The levels of ACE are found to depend on ID variant. In DD homozygotes the levels of ACE are almost a double than in II homozygotes [9] [12]. Although the D allele is linked to increase of plasma ACE activity in COPD, the D allele has not been associated with pulmonary hypertension or COPD, which is in accordance with our results [17].

**Figure 2:**
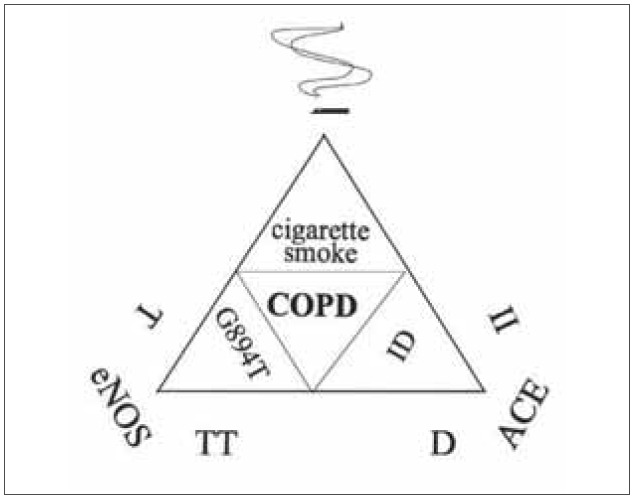
Interactions of the eNOS and ACE genes and cigarette smoking in chronic obstructive pulmonary disease. Figure 2 Gene-gene interaction between the eNOS TT and ACE D showed risk (OR=4.21, p=0.033) for the development of COPD. Carriers of double risk genotype, the eNOS TT and ACE DD were more frequent in COPD patients than in NLF and VBD controls (4.1% versus 0.0%). Gene-environment interactions between the eNOS 894T and cigarette smoking (OR=3.37, p=0.013) and between the ACE II and cigarette smoking (OR=4.21, p=0.009) showed risk for the occurrence of COPD.<br>ACE-angiotensin converting enzyme, eNOS-endothelial nitric oxide synthase, COPD-chronic obstructive pulmonary disease.

The present study is the first to demonstrate genegene and gene-environment interactions between the eNOS and ACE genes and cigarette smoking in the pathogenesis of COPD summarized in Figure 2.

Tagged by functional variants G894T and ID, eNOS and ACE double risk genotype (TT and DD) was identified with the frequency of 4.1% in patients, while no carrier was detected in NLF controls or VBD controls (Table 3) in our study. Likewise, gene-gene interaction between eNOS and ACE was identified when patients were compared with VBD controls (OR=4.21, p=0.033), Table 4.

For the first time, our study emphasizes potential risk, in carriers of double risk genotype of eNOS and ACE for the occurrence of COPD. None of the variant increases OR as a single risk factor, while when both are present, OR is significantly increased (Table 4). We suppose that common, low penetrance variants, such as G894T and ID, jointly operating could produce significant effect resulting in the development of complex disorders such as COPD, which also could be useful in prevention and choice of the pharmacological COPD treatment.

Interaction between eNOS and ACE has been described by Ahsan et al. [12] who noticed that the levels of plasma NO decrease from ACE II, ID to DD genotype, as well as the levels of plasma ACE increase from eNOS GG, GT to TT genotype. Thus, regarding gene-gene interaction identified in our study, carriers of the TT and DD genotype, would be expected to have an extremely low and high levels of NO and ACE, respectively. In mentioned study, a combination of the ACE II and eNOS GG genotypes was found to have protective effect on COPD, which is consistent with the results of our study that eNOS TT and ACE DD interaction represents risk for COPD. In another study the combination of eNOS 894T and ACE D variants was found to contribute to the genetic susceptibility to *Mycoplasma pneumoniae* pneumonia [30].

A link between eNOS and ACE is realized within the complex network of biochemical pathways (Figure 1). The simplest interaction is realized by caveolin-1, GUCY1A1 and BDKRB2. Bradykinin, that upon activating BDKRB2 receptor increases the expression and activity of eNOS exerting vasodilatory effects, is inactivated by ACE [16]. Thus, high levels of ACE and low levels of bradykinin and eNOS have unfavorable effect on endothelial function.

GUCY1A1, also known as GUCY1A3, is an effector enzyme of nitric oxide generated by eNOS, producing cyclic guanosine monophosphate leading to vasodilation, blood pressure lowering and inhibition of platelet aggregation [31]. On the other side, nitric oxide downregulates the synthesis of ACE [32]. Gene variants which inactivate the eNOS and GUCY1A1 genes are thought to have adverse effect on blood pressure, while an antihypertensive outcome is realized through inhibition of ACE activity and upregulation of GUCY1A1 expression [31].[33] Additionally, a GUCY1A1 stimulator has proven effective in the treatment of pulmonary hypertension [31].

In endothelial cells inactive state of eNOS is maintained through direct interaction with caveolin-1 [34]. The eNOS G894T variant contributes to endothelial dysfunction by diminishing eNOS and caveolin-1 interaction, altering eNOS caveolar localization and reducing the shear-dependent eNOS activation [27]. Caveolin-1 also regulates the expression of ACE in pulmonary endothelial cells [35]. Reduced Caveolin-1 expression was found in the vessels of COPD patients with pulmonary hypertension [36] [37].

Gene-environment interaction between eNOS 894T variant and cigarette smoking with a risk of 3.37 for COPD was identified in our study, Table 4. Endothelial dysfunction is not only an early feature of COPD, but is also seen in smokers without COPD, implicating the role of smoking in generation of initial endothelial injury. The expression of eNOS is reduced in pulmonary arteries of COPD patients with pulmonary hypertension and heavy smokers [28]. Endothelial dysfunction has been linked to reduced release of bioavailable NO. Cigarette smoking reduces generation of bioavailable NO [7]. Apart from all, oxidative stress can cause eNOS uncoupling and superoxide production with major consequences on endothelial function [38]. Accordingly, gene-environmentinteraction of the T allele, linked with down regulation of NO, and cigarette smoking, associated with endothelial dysfunction, identified in our study, can influence the pathogenesis of COPD by their joint injurious effect.

Interaction between the ACE II genotype and cigarette smoking with risk of 4.21 for the occurrence of COPD was identified for the first time in our study (Table 5). The I allele, associated with lower ACE levels, was not considered to be a risk factor. However, lung inflammation is commonly characterized by increase of chemotactic tripeptide proline-glycineproline (PGP), mostly acetylated by cigarette smoke. Acetyl-PGP is degraded by ACE, which limits the neutrophilic inflammation in the lungs of COPD patients and smokers [39]. The ACE II genotype is associated with the lowest levels of ACE, and AcPGP generated in smokers may be inefficiently degraded promoting additional lung inflammation. The study of Busquets et al. [40] reported that the DD genotype was associated with COPD in smokers. However, these results came from relatively small sample and different study design. The study of Shaw et al. [13] reported association of ACE I variant with lower FEV_1_ values in COPD smokers/exsmokers which is in accordance with our results.

Our results may also be relevant for prevention and therapy of COPD that should regard endothelial function protection by using eNOS enhancing compounds and non-ACE inhibitor antihypertensives [31] [38] [41].

In conclusion, a lack of records about pulmonary hypertension in COPD patients and small sample size are limitations of our study. However, consideration of the effects of interactions of the eNOS and ACE genes and cigarette smoke, identified between carefully selected patients with COPD and two control groups in a broader context brings important clues. Future studies on larger cohorts should provide more evidence of interactions between common genetic and environmental factors in COPD which could be useful in disease prevention and choice of therapy. Besides, further studies are needed to address the mechanisms of endothelial dysfunction related to the pathogenesis of COPD. Moreover, a novel gene-gene and gene-environment interactions, unrecognized up to now, were identified in our research as a possible cause of lung damage, contributing to »missing heritability« of COPD and ultimately improving our understanding of COPD pathogenesis.

## Dodatak

### Funding

This work was supported by the Ministry of Education, Science and Technological Development of Republic of Serbia under Contract 451-03-68/2022-14/200042.

### Disclaimer

AT, the views expressed herein represent those of the author and do not necessarily represent the views or practices of the authors’ employers or any other party.

### Conflict of interest statement

All the authors declare that they have no conflict of interest in this work.

### List of abbreviations 

ACE, angiotensin converting enzyme;<br>AGTR2, angiotensin II receptor type 2;<br>AKT1, AKT serine/threonine kinase 1;<br>ATP6AP2, ATPase H+ transporting accessory protein 2;<br>BDKRB2, bradykinin receptor B2;<br>CAV, caveolin;<br>CI, confidence interval;<br>COPD, Chronic obstructive pulmonary disease;<br>EDNRB, endothelin receptor type B;<br>EIF4G2, eukaryotic translation initiation factor 4 gamma 2;<br>eNOS, endothelial nitric oxide synthase;<br>FEV1, forced expiratory volume in 1 second;<br>FVC, forced vital capacity;<br>GUCY1A, guanylate cyclase 1 soluble subunit alpha;<br>GUCY1B, guanylate cyclase 1 soluble subunit beta;<br>HSP90AB1, heat shock protein 90 alpha family class B member 1;<br>LYPLA1, lysophospholipase 1;<br>MYH9, myosin heavy chain 9;<br>N-number of subjects;<br>NLF, normal lung function;<br>NOSIP, nitric oxide synthase interacting protein;<br>NOSTRIN, nitric oxide synthase trafficking;<br>NSMAF, neutral sphingomyelinase activation associated factor;<br>OR, odds ratio;<br>*p*, p value;<br>REN, renin;<br>SEM, standard error of mean;<br>SPSS, statistical package for the social sciences;<br>VBD, voluntary blood donors;<br>ZDHHC21, zinc finger, DHHC, type containing 21
